# 2101. CMV Infections in Multiple Myeloma Patients

**DOI:** 10.1093/ofid/ofac492.1723

**Published:** 2022-12-15

**Authors:** Sahrish Ilyas, Samantha E Jacobs, Meenakshi Rana, Emily Baneman

**Affiliations:** University of Pennsylvania, Philadelphia, Pennsylvania; Icahn School of Medicine at Mount Sinai, New York, New York; Icahn School of Medicine at Mount Sinai, New York, New York; Icahn School of Medicine at Mount Sinai, New York, New York

## Abstract

**Background:**

Substantial progress in the treatment of multiple myeloma (MM) has led to improved patient outcomes and prolonged survival of patients. These therapeutic advances have resulted in increased cumulative immunosuppression leading to increased susceptibility to opportunistic infections including CMV infection. The significance of CMV infection in MM patients is not well understood. We sought to describe the clinical characteristics and outcomes of patients with MM who develop CMV infection.

**Methods:**

Retrospective chart review of MM patients at Mount Sinai Hospital in NY who had CMV DNA PCR sent. The Mount Sinai Multiple Myeloma Database was utilized to identify patients who developed CMV viremia. Demographic, clinical and laboratory data were abstracted from electronic medical records. IRB approval was obtained.

**Statistical Analysis**

Factors associated with 30-day mortality by univariate analysis were evaluated using Fischer’s exact and Wilcoxon rank-sum test.

**Results:**

**Demographics**

414 MM patients had CMV PCR sent at least once. Forty-six cases of CMV infection were identified.

**CMV Infection Characteristics**

Forty-six patients were found to have CMV viremia, defined as PCR >500 IU/mL. Twenty-two (47%) had symptomatic infection. One patient had CMV pneumonitis and in 7/46 (15%) patients, CMV end-organ disease was suspected. Twenty (43%) patients had progressive disease status, 73% had prior history of autologous stem cell transplant and 85% had received steroids within 30 days of CMV infection. Twenty-nine (63%) patients had concurrent infections within 30 days of CMV infection, including bloodstream infection (n=16), bacterial pneumonia (n=9), respiratory viral infection (n=6), infectious colitis (n=5), and fungal infection (n=3).

**Outcomes**

9/46 (19%) patients died within 30 days of CMV infection and 12/46 (26%) required ICU admission during that hospitalization. In univariate analysis of risk factors associated with mortality, higher weekly steroid dose approached statistical significance (p=0.06) and peak CMV PCR >1000 IU/mL (p.008) was associated with lower mortality.

**Figure:**
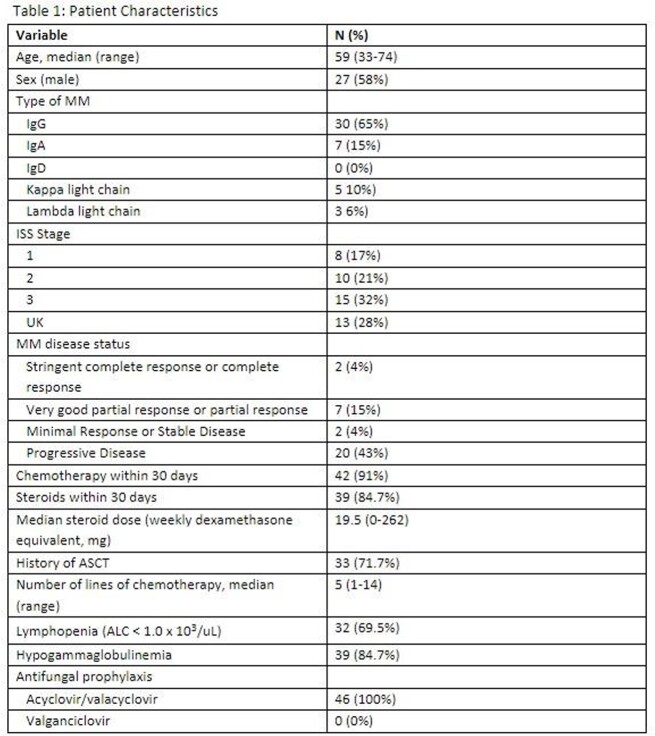

**Figure:**
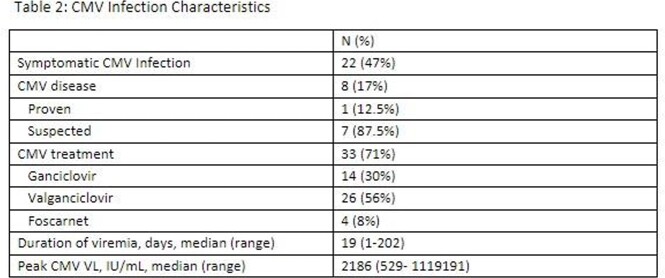

**Figure:**
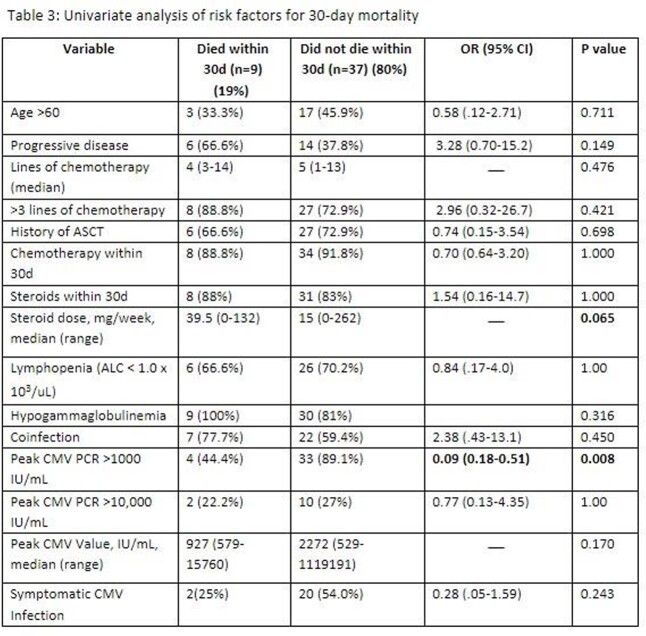

**Conclusion:**

CMV infection is associated with morbidity and mortality in MM patients. Prospective studies are needed to better assess the clinical significance of CMV reactivation in this population.

**Disclosures:**

**Emily Baneman, MD**, Merck: Grant/Research Support.

